# Substituent Control of σ-Interference Effects in the Transmission of Saturated Molecules

**DOI:** 10.1021/acsphyschemau.2c00016

**Published:** 2022-04-14

**Authors:** Marc H. Garner, Mads Koerstz, Jan H. Jensen, Gemma C. Solomon

**Affiliations:** † Nano-Science Center, University of Copenhagen, Universitetsparken 5, DK-2100 Copenhagen Ø, Denmark; ‡ Department of Chemistry, University of Copenhagen, Universitetsparken 5, DK-2100 Copenhagen Ø, Denmark

**Keywords:** Molecular Electronics, Destructive Quantum Interference, σ-Interference, Antiresonance, Single-Molecule Insulator, Silane, Substituent Effect

## Abstract

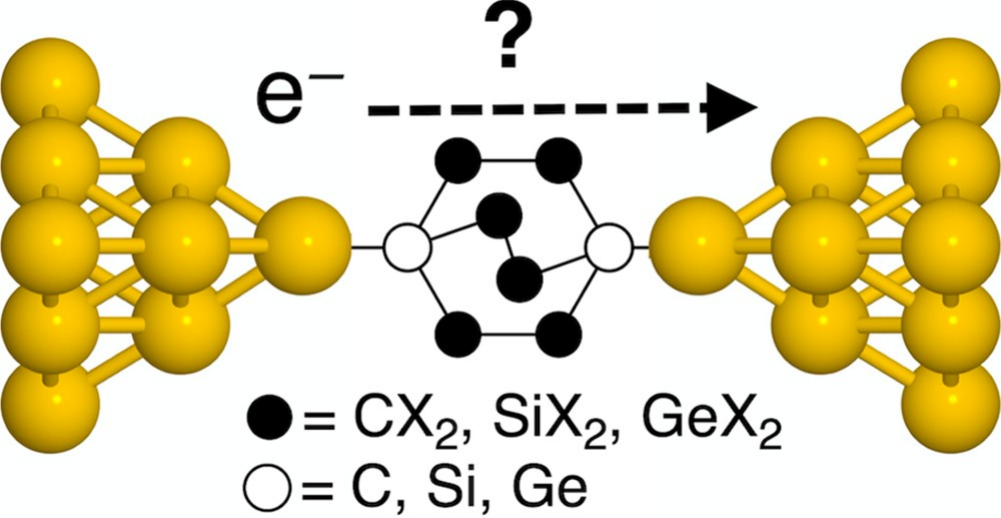

The single-molecule conductance of saturated molecules can potentially be fully suppressed by destructive quantum interference in their σ-system. However, only few molecules with σ-interference have been identified, and the structure–property relationship remains to be elucidated. Here, we explore the role of substituents in modulating the electronic transmission of saturated molecules. In functionalized bicyclo[2.2.2]octanes, the transmission is suppressed by σ-interference when fluorine substituents are applied. For bicyclo[2.2.2]octasilane and -octagermanes, the transmission is suppressed when carbon-based substituents are used, and such molecules are likely to be highly insulating. For the carbon-based substituents, we find a strong correlation between the appropriate Hammett constants and the transmission. The substituent effect enables systematic optimization of the insulating properties of saturated molecular cores.

Saturated organic molecules are considered to be good molecular insulators and find widespread use in studies of electronic and magnetic communication between functional molecular units.^1,2^ Destructive quantum interference in the σ-system can further enable the design of saturated molecules, where the electronic transmission is almost completely suppressed.^3−5^ Destructive σ-interference may appear in saturated molecules when all through-bond paths in the molecular backbone have at least one *gauche* defect, i.e., a dihedral angle approaching 0°.^3−8^ However, full suppression of the single-molecule conductance is not often achieved, and it is not always clear if the partial suppression is due to an interference effect.^5^ For example, the *gauche* conformations of simple linear alkanes have conductance lower than that of the *trans* conformations; however, clear signatures of destructive quantum interference are missing, such as sharp antiresonance dips in the electronic transmission.^8−16^ When σ-interference effects manifest, the conductance is highly sensitive to details in the molecular geometry. Based on the extensive topological understanding of interference effects in π-systems, we expect a similar intuition should be found for σ-systems.^17−21^ However, it is an ongoing challenge to understand the structure–property relationships for the single-molecule conductance of saturated group 14 systems.^9,22−27^

We reported a surprising chemical sensitivity of the σ-interference effect.^5^ Despite the benign nature of the substituents, the electronic transmission of functionalized cyclohexane, cyclohexasilane, bicyclo[2.2.2]octane, and bicyclo[2.2.2]octasilane is systematically lower when the molecules are permethylated. This is exemplified in Figure 1, where the Landauer transmission for Au–molecule–Au junctions of nonmethylated (H) and permethylated (Me) bicyclo[2.2.2]octasilane (Si222) is shown along with that of the equivalent linear tetrasilane (Si4) molecules. Although both Si222-H and Si222-Me show clear suppression of the transmission compared with their linear counterparts Si4-H and Si4-Me. Both linear systems have similar transmission regardless of the choice of substituents. The transmission of Si222-Me is significantly lower than that of Si222-H and furthermore shows a clear antiresonance near the Fermi energy. This difference between Si222-Me and Si222-H is clearly a substituent effect as the change to the molecular core is almost negligible.^5^

**Figure 1:**
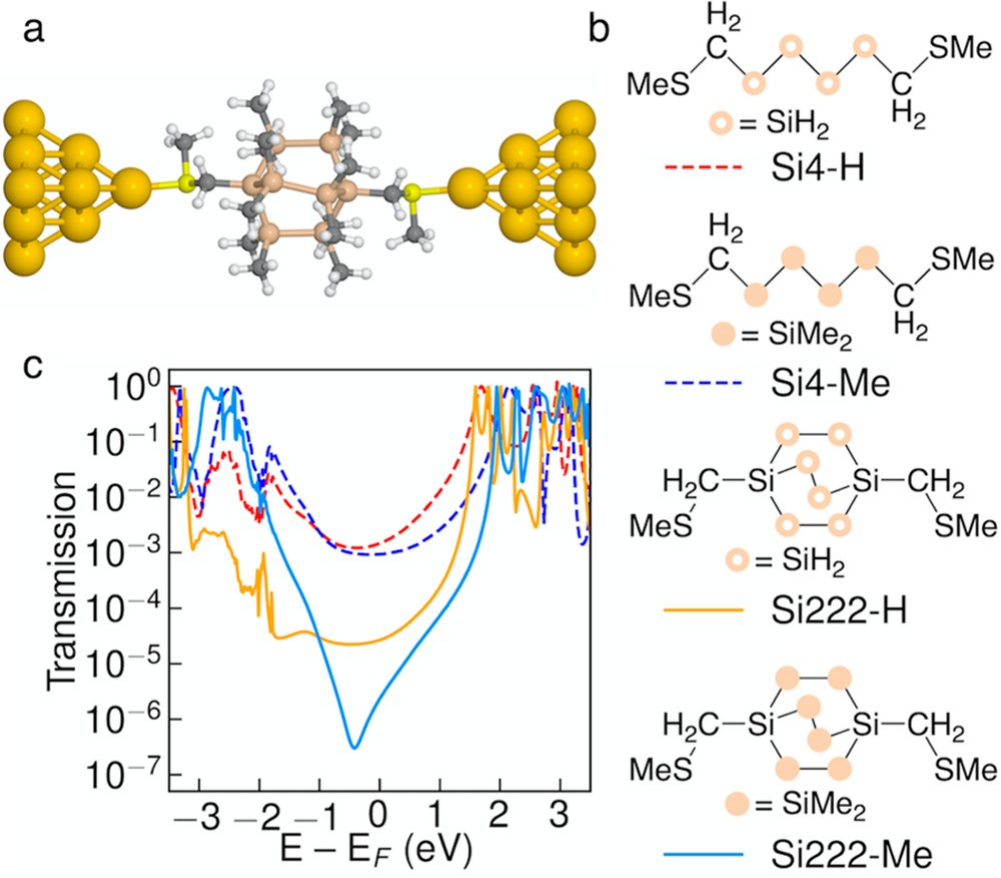
Transmission of methylthiomethyl-functionalized nonmethylated (H) and permethylated (Me) tetrasilane (Si4) and bicyclo[2.2.2]octasilane (Si222). (a) Optimized junction structure of Si222-Me. (b) Overview of the four molecules. (c) Transmission plotted semilogarithmically against energy for Si4-H, Si4-Me, Si222-H, and Si222-Me.

Substituent effects are well-established for destructive quantum interference effects in π-conjugated molecules.^28−31^ The destructive π-interference effect can be systematically manipulated depending on the electron-donating or -withdrawing character of the substituent.^32−40^ Here, we examine the substituent effect on the electronic transmission of methylthiomethyl-functionalized bicyclo[2.2.2]octane (C222), bicyclo[2.2.2]octasilane (Si222), and bicyclo[2.2.2]octagermane (Ge222). Fully X-substituted C222-X, Si222-X, and Ge222-X structures are sampled using RDKit, as described in the Supporting Information part A.^41^ Common to all the methylthiomethyl-functionalized bicyclo[2.2.2] systems, the molecular core is chiral but has no conformational freedom. Therefore, the orientations of the two linkers relative to each other provide three conformations. These are shown in Figure 2 as a Newman projection along the bridgehead axis of the molecule and as the optimized conformations of Si222-H. The Si–Si–CH_2_–S dihedral angles of the linkers are rotated to sample the three conformations. In accordance with previous work, we refer them as the *anti*, *ortho*, and *cis* conformers. The energy difference between these three is small with few exceptions, and the conformations will not be distinguishable at room temperature. We sample these three conformations in all studied molecules; their energies are listed in Table S1. For a control system, we examine the equivalent linear molecule in its all-*trans* conformation, as shown in bottom panel of Figure 2.

**Figure 2:**
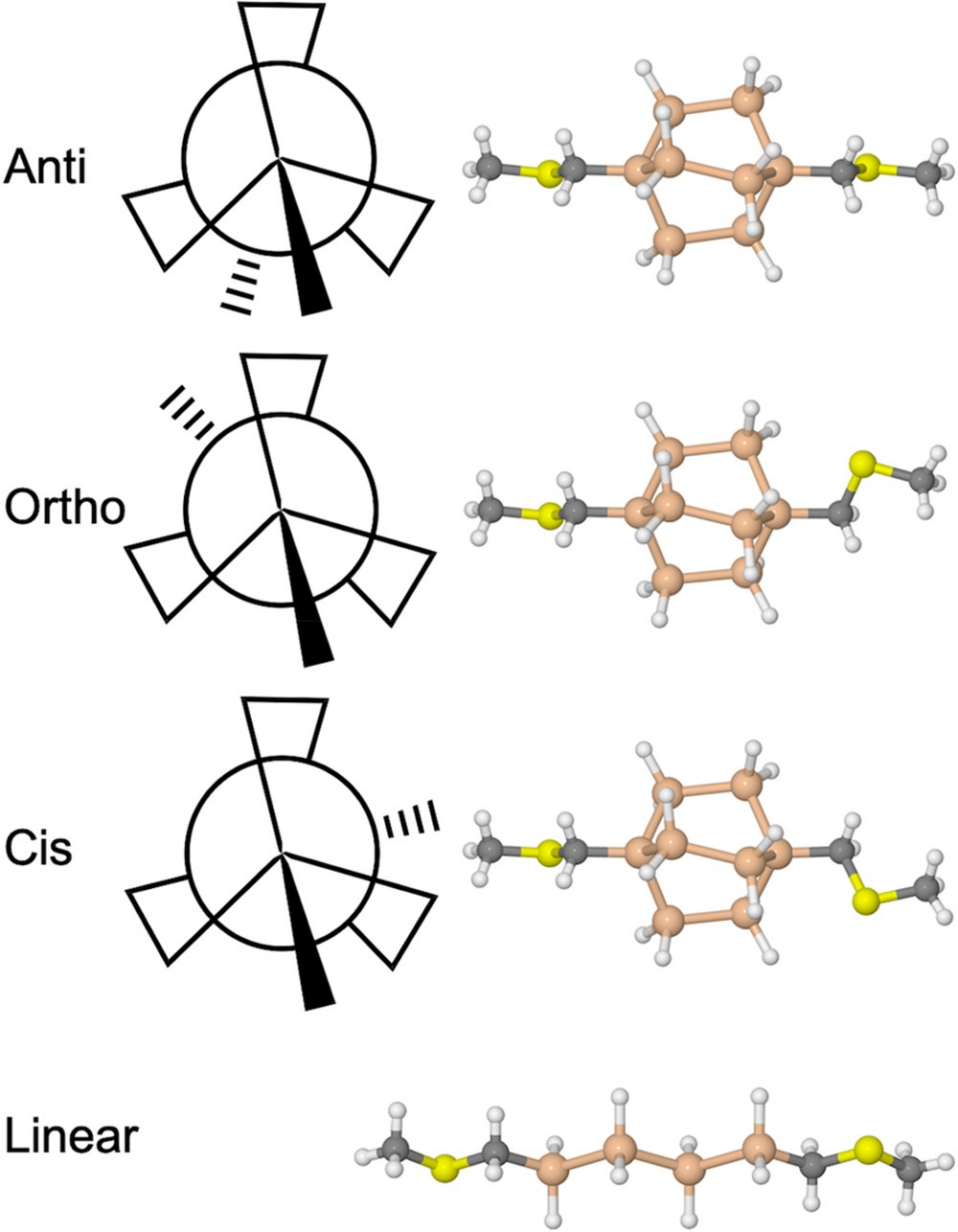
Three generic conformations of methylthiomethyl-functionalized C222-X, Si222-X, and Ge222-X molecules and all-*trans* linear control molecule. Left: Newman projection along the axis of the bridgehead atoms of the bicyclic cage. Right: Corresponding optimized structures of Si222-H and linear Si4-H.

All structures are optimized using density functional theory (DFT) as implemented in Gaussian09 using the PBE exchange-correlation (XC) functional with the 6-311G(d,p) basis set.^42,43^ Single-molecule junctions were made by placing the optimized molecules between two four-atom Au pyramids on 5 × 5 Au fcc(111) surfaces. The initial junction structure is built using a Au–S bond length of 2.5 Å and a Au–S–C bond angle of 110°. The terminal methyl groups are rotated to a dihedral angle of ±90° in order to make space for the Au atoms in the transoid position (±170°).^44^ Junctions are relaxed to a force threshold of 0.06 eV/Å using DFT with the PBE XC functional and double-ζ plus polarization basis set as implemented in ASE and GPAW.^43,45−47^ Finally, the Landauer transmission is calculated using the NEGF approach as implemented in ASE.^45^ The Fermi energy is computed ab initio in the junction structure that includes four layers of gold atoms with periodic boundary conditions to model the bulk properties of each electrode.

We devise an extensive list of substituents based on the criterion of some extent of chemical realism, though in some cases also on chemical curiosity. The different substituents on C222-X, Si222-X, and Ge222-X are to some extent limited by the size of the molecular frame, and therefore, not all substituents can be used. Scheme 1 provides an overview of all 34 molecules studied. Optimizations of cyano-substituted Ge222-CN did not converge. It is a molecule we do not expect to be experimentally realistic, but it would have been of interest because CN is a very strong electron acceptor.

**Scheme 1:**
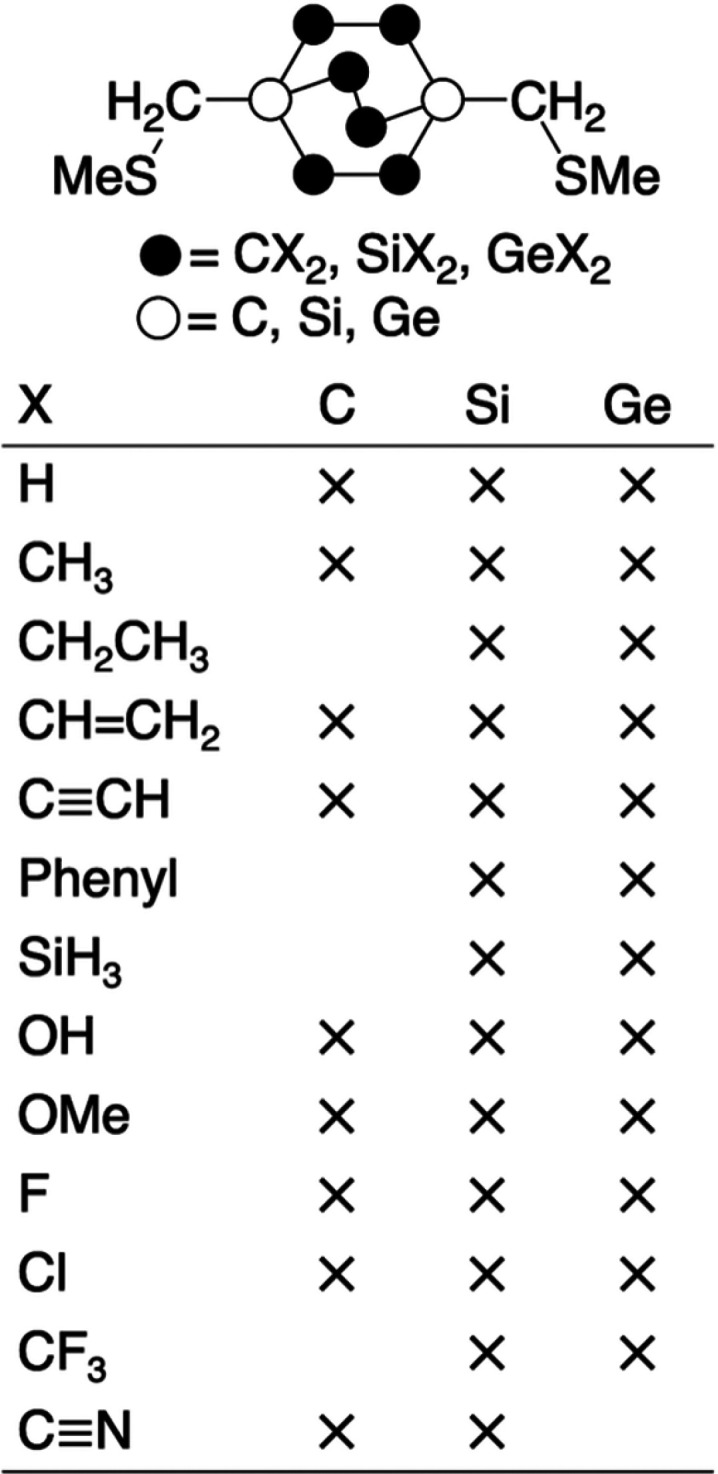
Overview of Substituents in C222-X, Si222-X, and Ge222-X

In the low-bias regime, we study here an insulating molecule that must have strong suppression of transmission near the Fermi energy in all energetically relevant conformers (see Table S1). The constrained structure of the bicyclo[2.2.2]octane frame makes them shorter than the equivalent butane motif molecules (methylthiomethyl-functionalized C4-X, Si4-X, and Ge4-X). Despite being shorter molecules (shorter tunneling barriers), previously studied bicyclo[2.2.2]octane systems have a transmission similar to or lower than that of their linear counterparts as a testament to their superior insulating properties.^3−5,25,48^

The transmission is plotted against energy for select Si222, Ge222, and C222 molecules in Figure 3. Transmission plots for all 102 structures are included in Supporting Information part C. As shown in Figure 3a, there is clear suppression of the transmission of Si222-Me and Si222-Ph due to deep antiresonances near the Fermi energy, with little dependence on the molecular conformation. There is also effective suppression of the transmission in Ge222-Me and Ge222-Ph (Figure 3b). Although the frontier orbitals are similar in the three conformers of Si222-Me and Ge222-Me (Figures S5 and S6), the transmission varies depending on the conformation in Ge222-Me. The transmission of the lowest Ge222-Me conformation (*cis*) is higher than that in the equivalent Si222-Me. Germanes were previously found to have conductance systematically higher than that in silanes,^49,50^ which is in agreement with increased σ-conjugation down group 14.^51−53^ However, we do not find this to be a systematic trend when comparing the equivalent Si222 and Ge222 systems with various degrees of destructive interference. Phenyl substituents may offer conductance for germanium slightly lower than that for methyl (Figure 3b). Similar suppression and variation are seen in the ethyl-, vinyl-, and ethynyl-substituted cases of Si222 and Ge222 (Figures S3 and S4). Carbon-based substituents thus seem particularly promising for fine-tuning the insulating properties of Si222 and Ge222 systems. Although alkyl and phenyl substituents are quite benign from a chemical point of view, they are particularly promising because they are realistic synthetic targets for silicon- and germanium-based molecular cores.^54−62^ We hope these results will motivate studies of the properties of substituted cyclic and bicyclic silanes and germanes.

**Figure 3:**
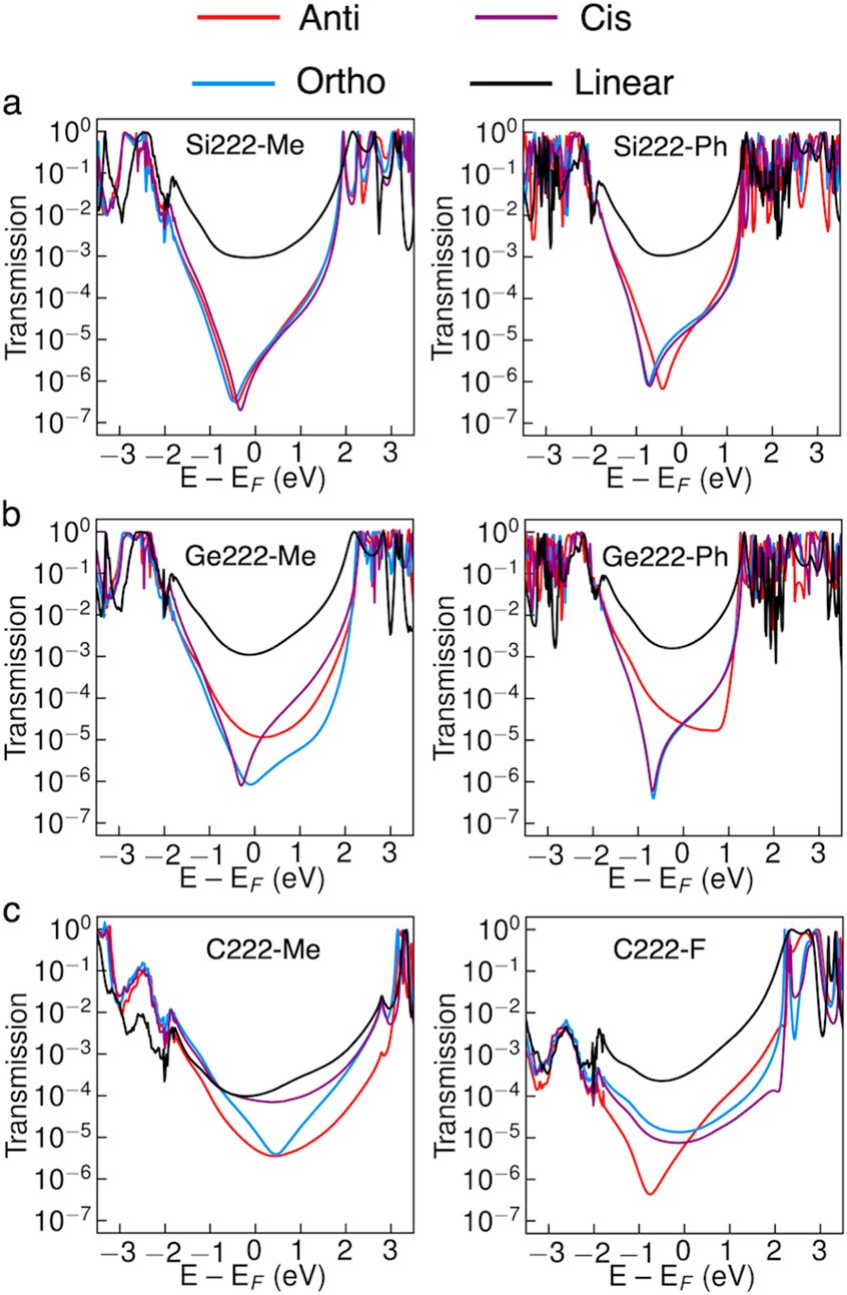
Transmission plots of methyl- and phenyl-substituted Si222-X (a) and Ge222-X (b) and methyl- and fluoro-substituted C222-X (c). These are compared with the equivalent linear silane, germane, and alkane, Si4-X, Ge4-X, and C4-X.

The transmission suppression is less clear in the C222 systems than for the silanes and germanes. A shown in Figure 3c, the transmission of C222-Me and C222-F does have antiresonances for some conformers. However, looking broadly at the C222 systems in Figure S2, it appears there are few cases with σ-interference. We note that the transmission of linear alkanes is much lower than that of linear silanes and germanes, in general,^50^ and the suppression effect is thus quite small in C222. For example, the suppression of the transmission in C222-H is suppressed by an order of magnitude compared to that of C4-H, in good agreement with experimental results.^5,48^ Peralkylated bicyclo[2.2.2]octanes are not the most realistic synthetic targets, whereas fluorinated alkanes make for more realistic targets for single-molecule junction studies.^63−65^ The transmissions of all C222-F conformers are suppressed over a broad energy range compared with those of C4-F. In agreement with previous work,^5^ the phase pattern of the HOMO and HOMO–1 differ for molecules with transmission suppression compared to the systems with less suppression (cf. C222-F and *ortho* conformation of C222-Cl, Figures S7–S9). Of the substituents studied for C222, fluorinated bicyclo[2.2.2]octanes are thus an interesting prospect for organic interference-based single-molecule insulators. They are still not as promising as the Si- and Ge-based systems as only the anticonformer has a deep antiresonance near the Fermi energy, whereas all three conformers are near-degenerate and are expected to be equally populated at room temperature (Table S1).

Certain substituents seem to promote the insulating properties of the three molecular cores. We further assess substituents based on their electron-withdrawing and -donating properties. Such effects have been classified in the extensive physical organic chemistry literature.^66^ In Figure 4a, the transmission at the Fermi energy is plotted for all substituted molecules sorted by their often used Hammett constants σ_p_, which are based on the substituent effect in *para*-substituted benzenes.^66^ Based on Figure 4a, the substituent effect is similar across the C222, Si222, and Ge222 series in some cases, predominantly carbon-based substituents (blue), whereas it differs in others. However, there is no clear correlation across the full transmission data set when plotted against σ_p_.

**Figure 4:**
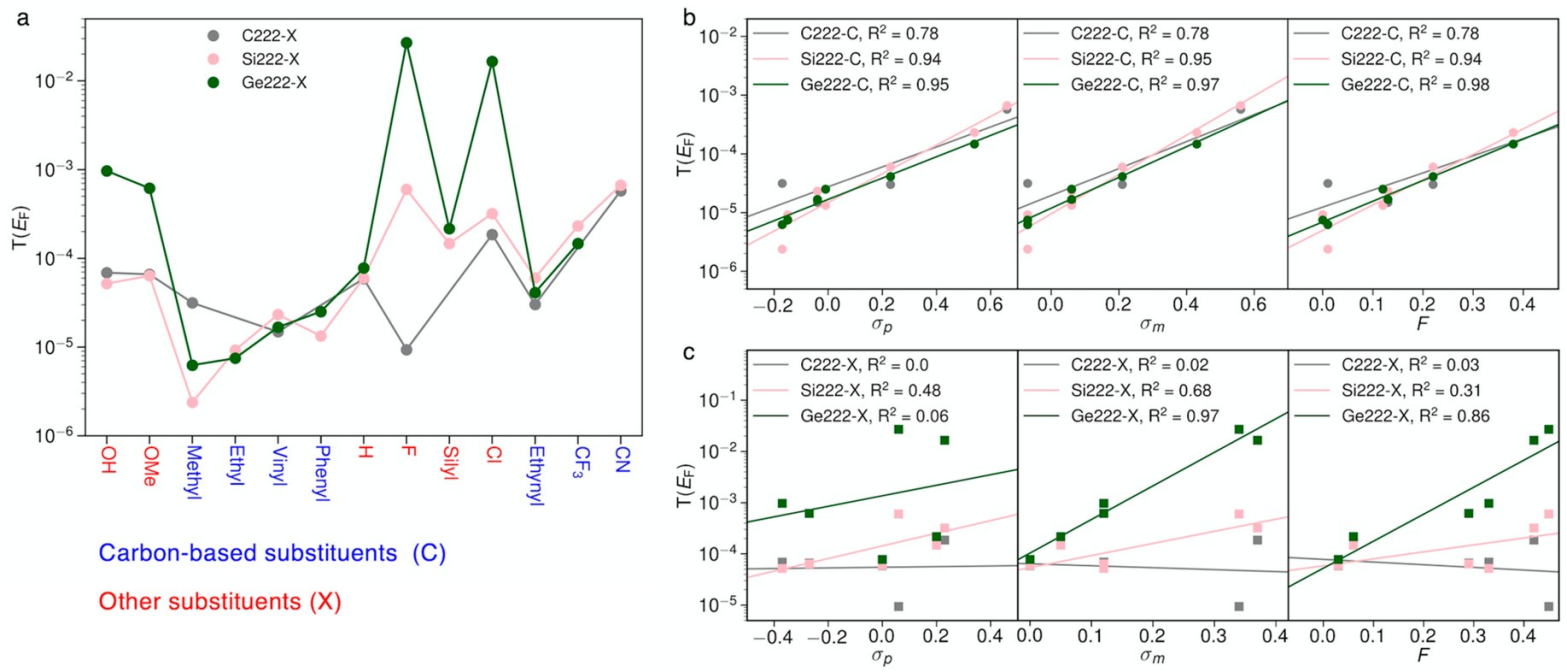
(a) Transmission at the Fermi energy plotted against substituents organized from donor to acceptor by *para* Hammett constants, σ_p_.^66^ (b) Transmission at the Fermi energy plotted as a function of the *para* and *meta* Hammett constants, σ_p_ and σ_m_, and as a function of the inductive parameter, *F*, for molecules with carbon-based substituents. (c) Same as in panel (b) for molecules with the remaining non-carbon-based substituents. Each data point is averaged over the three conformations. *R*^2^ values are provided for linear least-squares fit.

Motivated by the strong transmission suppression seen for many of the carbon-based substituents, we separate the data set into two types of substituents: those based on carbon (blue in Figure 4a) and the remaining that are not carbon-based (red in Figure 4a). We search further for quantitative correlations in substituent parameters that are determined in systems with suppressed or separated π-conjugation.^66^ In addition to σ_p_, we plot the transmission values against the Hammett constant, σ_m_, which are determined on *meta*-substituted benzenes and the inductive parameter *F*. Whereas all of these parameters are determined empirically, *F* may provide a better measure of the substituent effect on the σ-system as it uses a bicyclo[2.2.2]octane compound for reference.^66^ A shown in Figure 4b, the transmission of the three cores with carbon-based substituents is plotted against the three different Hammett constants. Although there is a weak correlation for C222, both Si222 and Ge222 transmission show strong correlation with all three empirical parameters. These correlations are consistently weaker for the non-carbon substituents as plotted in Figure 4c. There is some correlation for these substituents in the Ge222 with σ_m_ and *F*, but in the remaining systems, there is no correlation between the transmission and the Hammett constants.

The carbon-based substituents (listed in blue in Figure 4a) stand out from their non-carbon counterparts. They enable the lowest transmission for the Si222 and Ge222 systems, and their transmission correlates with the electron-withdrawing properties of the substituents as given by the empirical Hammett constants. These correlations are quite strong compared to the correlations reported in other single-molecule conductance studies.^67−69^ Carbon-based substituents thus provide an opportunity for fine-tuning σ-interference effects in silicon- and germanium-based compounds.

The carbon-based substituents we find here appear to be special for the silicon- and germanium-based cores but do not systematically promote interference effects in the carbon-based core. As previous studies have suggested, the σ-interference effect is highly sensitive to several structural and electronic parameters.^3−7,24^ In particular, cisoid dihedral angles are a requirement, which is fulfilled by all of the molecules we study here. We have tested for correlations with structural parameters in our data set but have not found any clear correlation (see Supporting Information part B for details). This indicates that the effect of the substituents is electronic (donor/acceptor effect) rather than from the distortion of the structure in the molecular core. While these two effects (electronic and structural) are not independent of each other, the clear correlation we see for the carbon-based substituents with the Hammett constants suggests the effect is primarily electronic. Most likely, carbon-based substituents are special because they tune the electronic structure within an ideal range where there is σ-interference in the transmission. This balance of parameters that enables interference effects in saturated molecules is still not well-described, but clearly the σ-interference effect is very sensitive to both chemical and structural changes.

To summarize, a wide range of substituents can result in significant destructive interference effects in the transmission of Si and Ge cores. These are substituents based on carbon, such as alkyl and phenyl substituents, and we show that this substituent effect is systematic by correlating the transmission with different Hammett constants. In the C cores, we find a number of instances where some combination of substituent and conformation exhibits an interference feature; however, variation with conformation is much more significant than in the Si and, to a lesser extent, Ge cores. There are no cases for the C cores where all conformations exhibit the uniform interference effects we see for Si. We find that fluorination appears to be the only substitution pattern that is likely to result in significant conductance–suppression in the carbon-based bicyclo[2.2.2]octane we have studied here, although even this does not produce destructive interference in all of the conformations studied but simply flattened transmission in two cases. σ-Interference thus appears to be much more sensitive to chemical changes than the more well-established π-interference effect. Until recently, we may have thought that saturated molecules are some of the simplest and best understood systems in molecular electronics. Recent results by us and other groups suggest there is much more potential,^5,70^ which may be applied for novel chemical designs. This letter is a step in this direction, and future work must continue to build on the electron transport structure–property relationship of saturated carbon molecules and their heavier group 14 analogues.

## Supporting Information Available

Sampling of molecular space; correlation between structure and transmission; transmission plots; frontier molecular orbitals (PDF)
Optimized molecular structures and junctions (ZIP)
