# Supplementary material for: Substituent Control of σ-Interference Effects in the Transmission of Saturated Molecules
Source: ACS Phys Chem Au. 2022 Apr 14;2(4):282–8. doi: 10.1021/acsphyschemau.2c00016 (PMC9955259; doi:10.1021/acsphyschemau.2c00016)
Supplement: Supplementary file 1 — pg2c00016_si_001.pdf [file pg2c00016_si_001.pdf]

Supporting Information for: Substituent Control of σ-interference Effects in the Transmission of Saturated Molecules

Marc H. Garner†[1,2], Mads Koerstz[1], Jan H. Jensen[1], Gemma C. Solomon[1,2]*

[1]Nano-Science Center, University of Copenhagen, Universitetsparken 5, DK-2100 Copenhagen Ø, Denmark.

[2]Department of Chemistry, University of Copenhagen, Universitetsparken 5, DK-2100 Copenhagen Ø, Denmark.

***gsolomon@chem.ku.dk**

Table of Contents

A. Sampling the molecular space of C222-X, Si222-X, and Ge222-X……………….2

B. Correlation between structure and transmission………………………………….4

C. Transmissions of C222-X, Si222-X, and Ge222-X…………………………………6

D. Frontier Molecular Orbitals………………………………………………………9

References…………………………………………………………………………….12

A. Sampling the molecular space of C222-X, Si222-X, and Ge222-X

Structures for all fully substituted C222-X, Si222-X, and Ge222-X were generated using an RDKit-script.[1] For each fully substituted motif, we created 100 random conformers while constraining the core bicyclo[2.2.2]-unit to the optimized structures of the unsubstituted parent molecule we found in previous work.[2] Thus, only the ligand conformers are sampled. The appropriate ligand conformations were selected by choosing the low energy structure based on the UFF energy.[3-4] The linker dihedral angles in both ends are set to 180° creating the anti conformer. Hereafter, the cis and ortho conformers were generated by systematically rotating one end by ±120°, yielding the three general conformers for each of the substituent motif.

The script is available online at https://github.com/jensengroup/substituent_insulater_screening.

All DFT-optimized structures and junctions are available online as supporting files. Energies relative to the most stable conformation is included in Table S1.

Table S1: Energy (eV) of conformers relative to most stable conformer (PBE/6-311G(d,p))

	Anti	Cis	Ortho
C222_H	0.011	0.003	0.000
C222_Me	0.022	0.000	0.015
C222_vinyl	0.000	0.024	0.022
C222_ethynyl	0.010	0.002	0.000
C222_OH	0.000	0.325	0.286
C222_OMe	0.000	0.209	0.009
C222_F	0.003	0.003	0.000
C222_Cl	0.007	0.019	0.000
C222_CN	0.029	0.000	0.006
Ge222_H	0.015	0.010	0.000
Ge222_Me	0.000	0.017	0.021
Ge222_Et	0.037	0.000	0.077
Ge222_vinyl	0.000	0.026	0.016
Ge222_ethynyl	0.008	0.000	0.001
Ge222_phenyl	0.040	0.001	0.000
Ge222_SiH3	0.032	0.003	0.000
Ge222_OH	0.042	0.119	0.000
Ge222_OMe	0.000	0.005	0.012
Ge222_CF3	0.032	0.000	0.009
Ge222_F	0.000	0.023	0.031
Ge222_Cl	0.018	0.013	0.000
Si222_H	0.009	0.004	0.000
Si222_Me	0.000	0.002	0.001
Si222_Et	0.048	0.028	0.000
Si222_vinyl	0.037	0.063	0.000
Si222_ethynyl	0.012	0.000	0.002
Si222_phenyl	0.031	0.002	0.000
Si222_SiH3	0.040	0.000	0.009
Si222_OH	0.116	0.006	0.000
Si222_OMe	0.000	0.052	0.061
Si222_F	0.004	0.000	0.000
Si222_Cl	0.012	0.000	0.000
Si222_CN	0.028	0.011	0.000
Si222_CF3	0.046	0.000	0.006

B. Correlation between structure and transmission

Electronic substituent effects may be accompanied by a structural deformation of the molecule. It is clear from previous studies that the transmission can be quite sensitive to structural changes when there are quantum interference effects.[5-8] Rather than a direct electronic substituent effect, it is possible that structural deformation due to the substituents may be responsible for the large variations we see in the transmission. In Figure S1, we explore the possible correlation between key structural parameters and the transmission. We plot the transmission of the Fermi energy for all **C222**, **Si222**, and **Ge222** junctions separated by substituent type against geometrical parameters of the specific junction conformation. There are several bond lengths, bond angles, and dihedral angles which may be important. Plotted in Figure S1a and S1b, we find that the bridgehead distance is a suitable overall measure of the deformation of bond lengths and bond angles in the bicyclic cage structure. First, we note that the deformation is smallest for carbon and largest for germanium, which is to be expected based on the bonds generally becoming less rigid down the periodic group. There is no correlation for the **C222** systems while we do see a weak correlation for the silicon and germanium systems based on a linear fit to the data, both with carbon-based and non-carbon substituents. The bridgehead distances are among the longest in the high transmission cases of silicon and germanium systems, which include the high-transmission cases with halide substituents.

The transmission at the Fermi energy is plotted against average of the three bridging dihedral angles, C-C-C-C, Si-Si-Si-Si, and Ge-Ge-Ge-Ge, for each junction structure in Figure S1c and S1d. This is a parameter that the transmission is known to be particularly sensitive to.[5,8] However, there is little correlation for any of the compounds studied here. Clearly, the dihedral angle is not the controlling geometrical parameter in molecules based on the bicyclo[2.2.2]octane-motif.

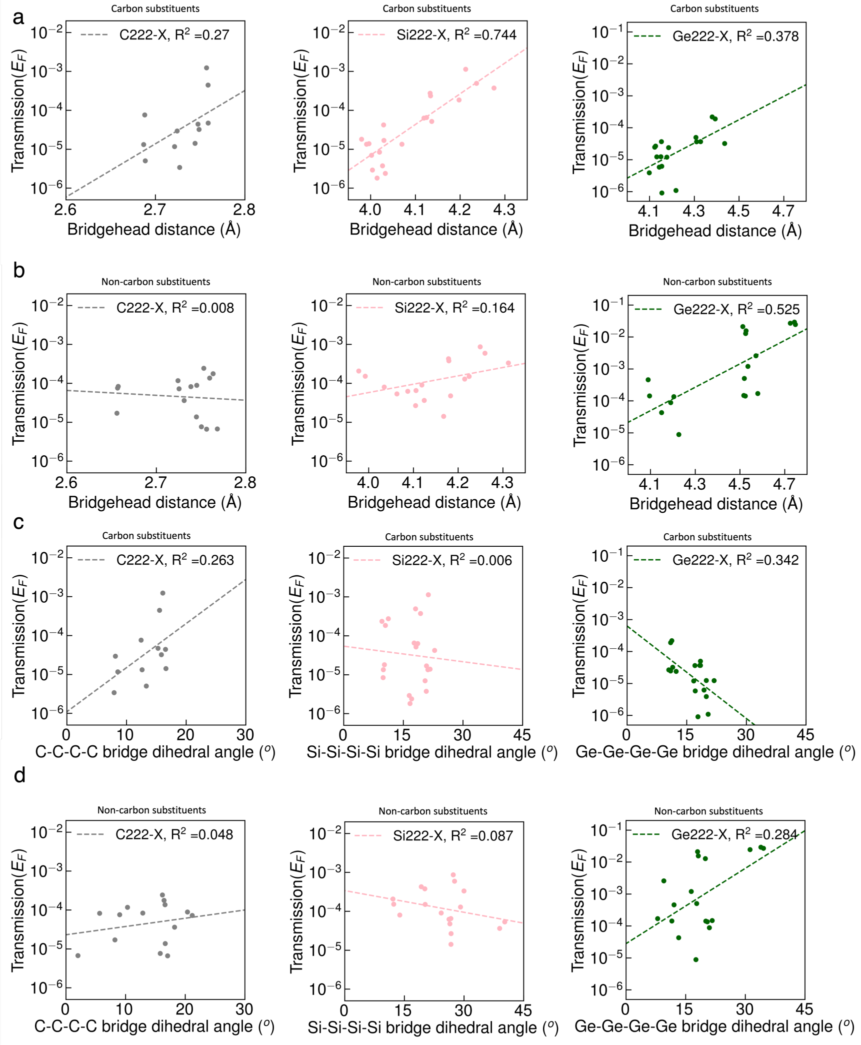

Figure S1. Transmission at the Fermi energy for all **C222**, **Si222**, and **Ge222** molecules plotted against structural parameters. a) Direct bridgehead C-C, Si-Si, or Ge-Ge distance of the bicyclic cage structure with carbon-based substituents. b) Direct bridgehead C-C, Si-Si, or Ge-Ge distance of the bicyclic cage structure with non-carbon substituents. c) Dihedral angle bridging the bicyclic cage with carbon-based substituents. d) Dihedral angle bridging the bicyclic cage with carbon-based substituents Each value is taken as the average of the three bridges forming a bicyclo[2.2.2]octane structure. R^2-values are provided for linear least-squares fit.

C. Transmissions of C222-X, Si222-X, and Ge222-X

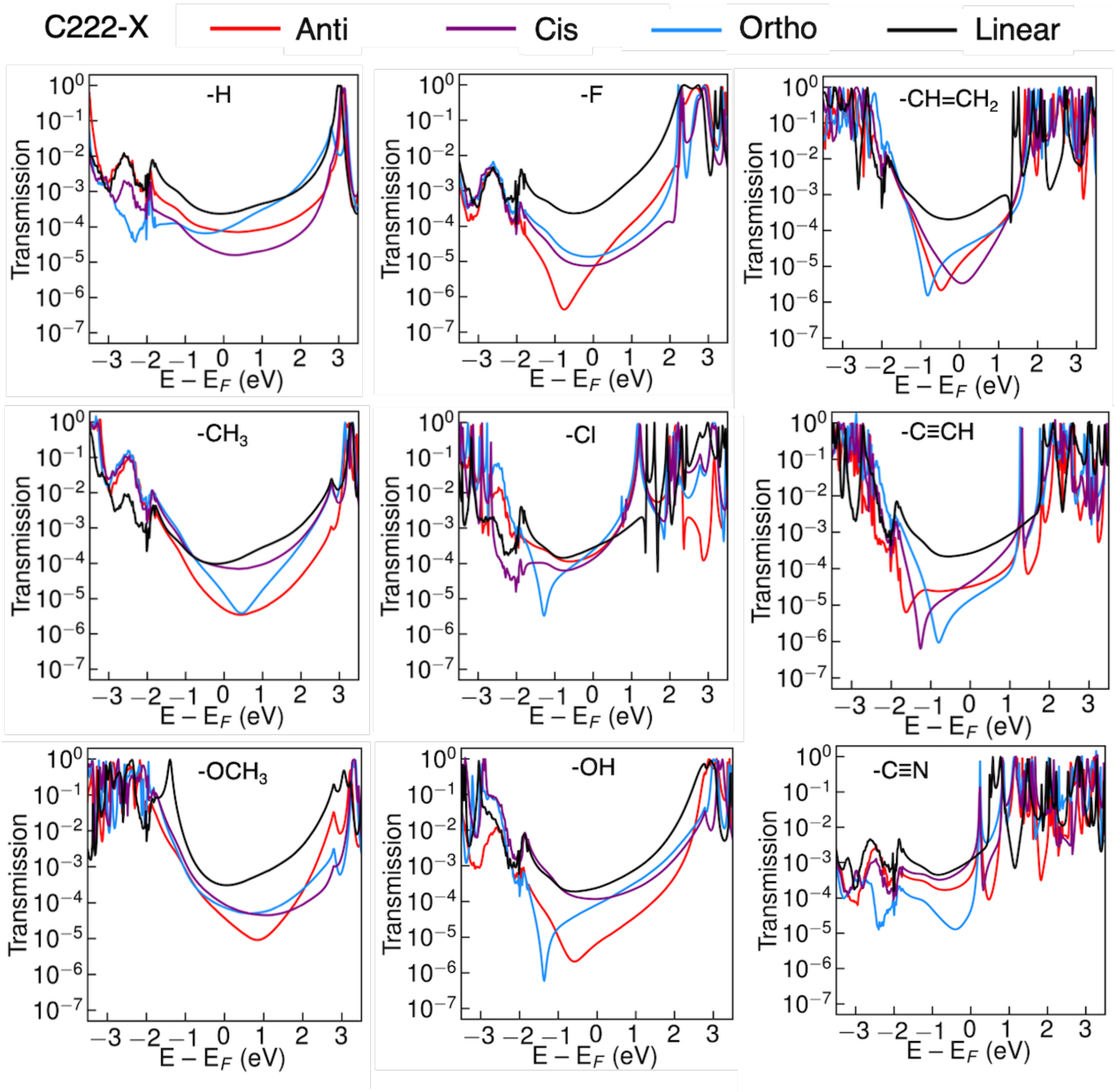

Figure S2. Transmissions of all substituted C222-X molecules, and their linear counterparts.

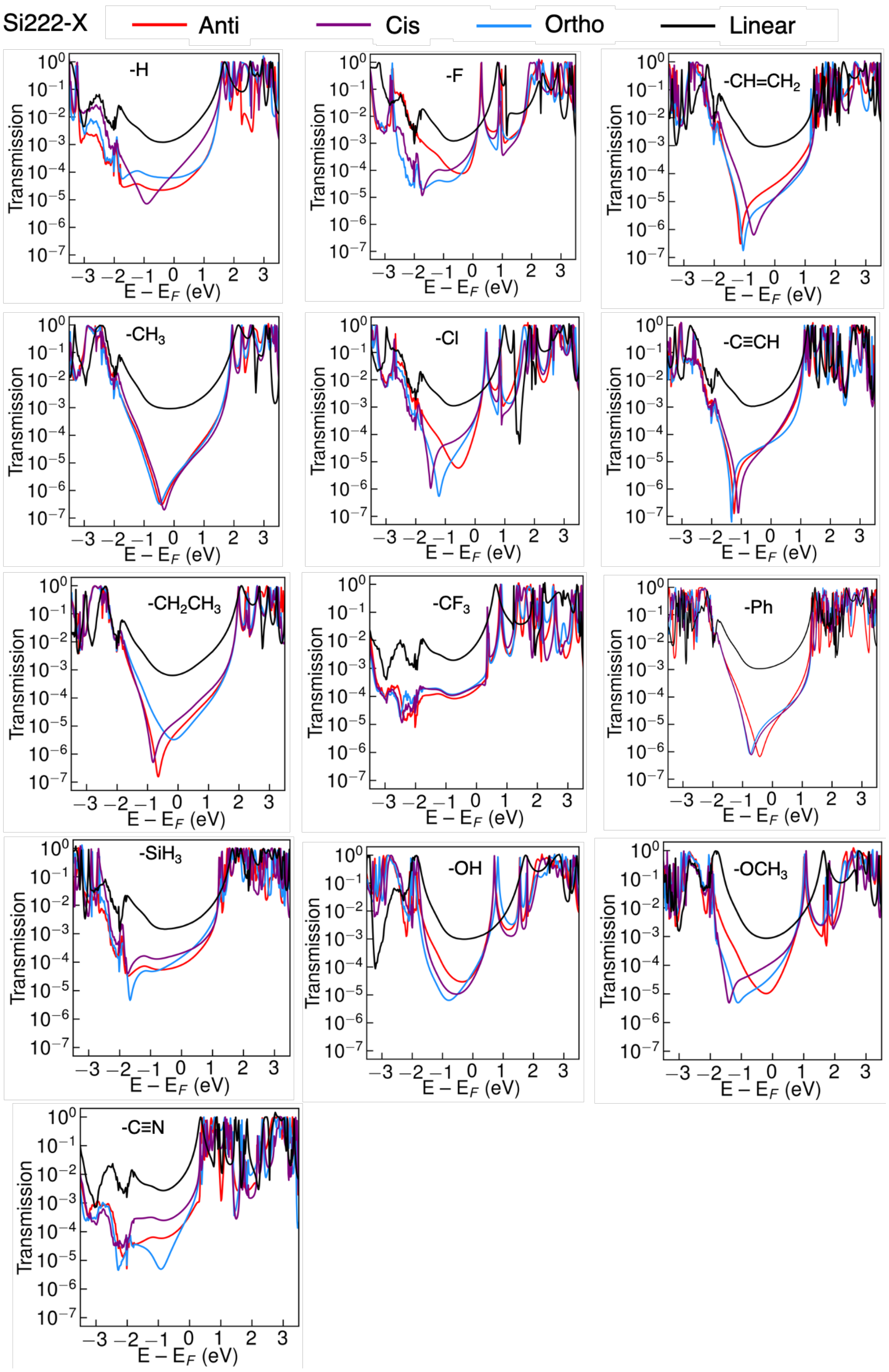

Figure S3. Transmissions of all substituted Si222-X molecules, and their linear counterparts.

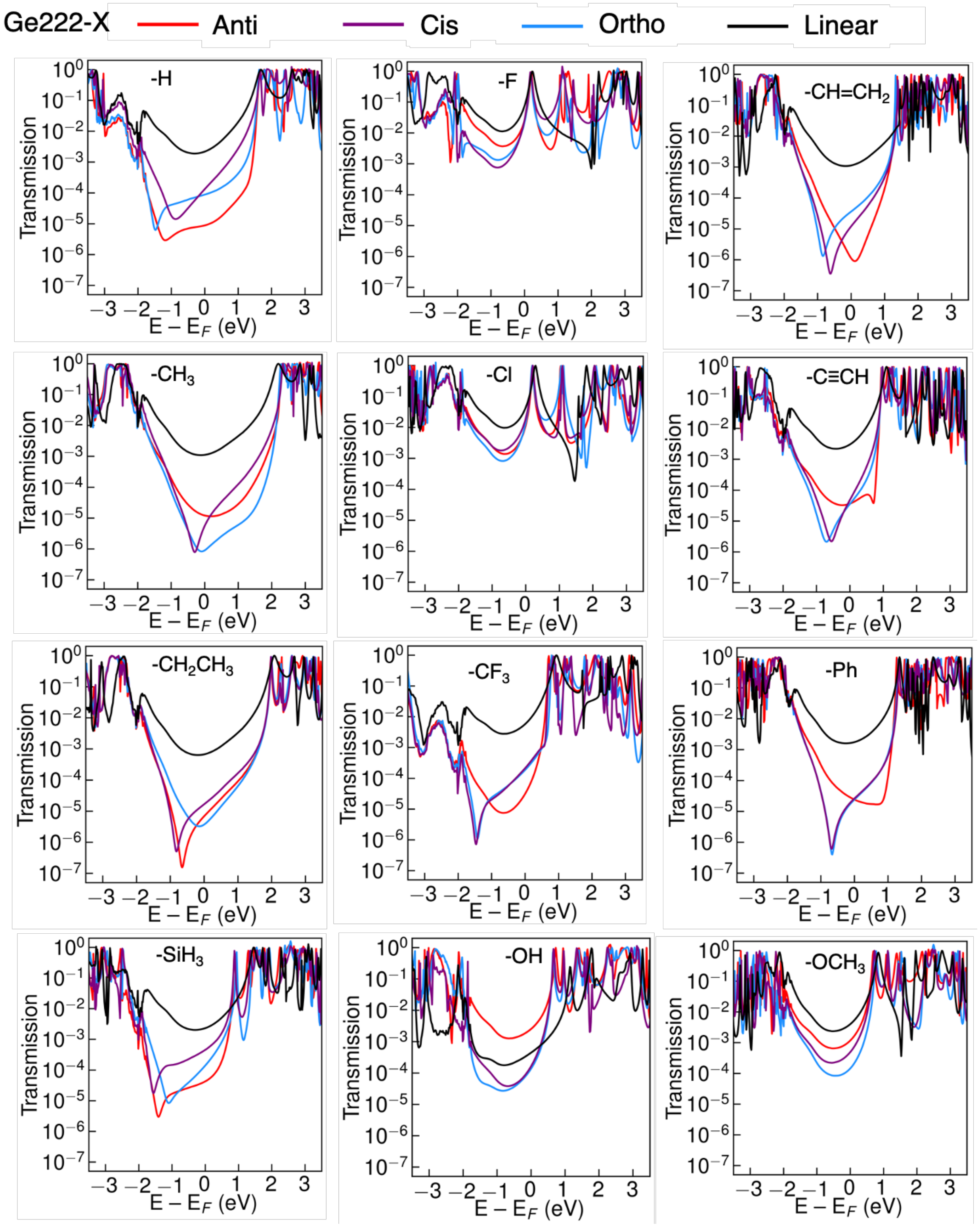

Figure S4. Transmissions of all substituted Ge222-X molecules, and their linear counterparts.

D. Frontier Molecular Orbitals

Frontier molecular orbitals from select molecules of the manuscript are plotted with isovalue = 0.05 using Avogadro.[9-10]

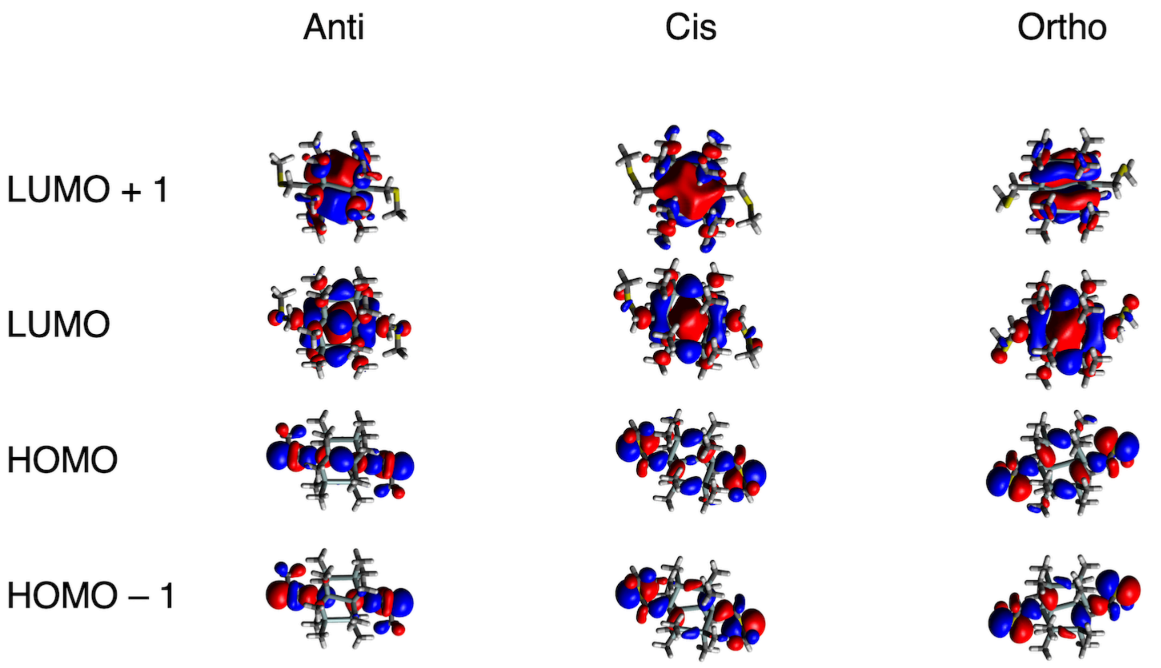

Figure S5. Frontier MOs of Si222-Me.

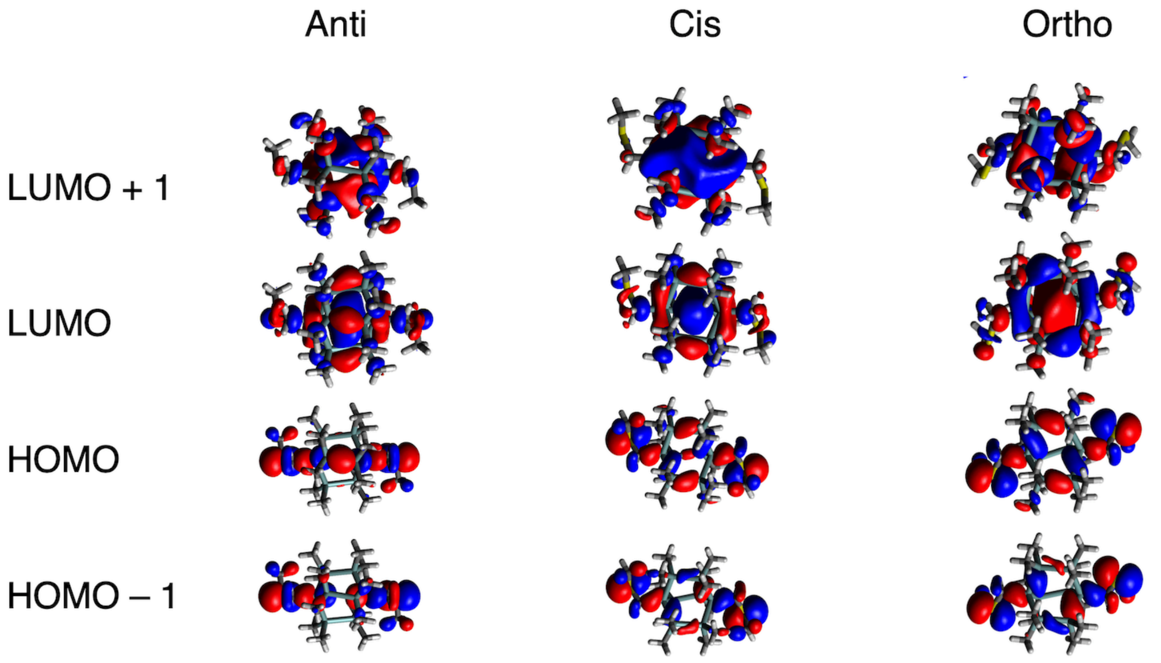

Figure S6. Frontier MOs of Ge222-Me.

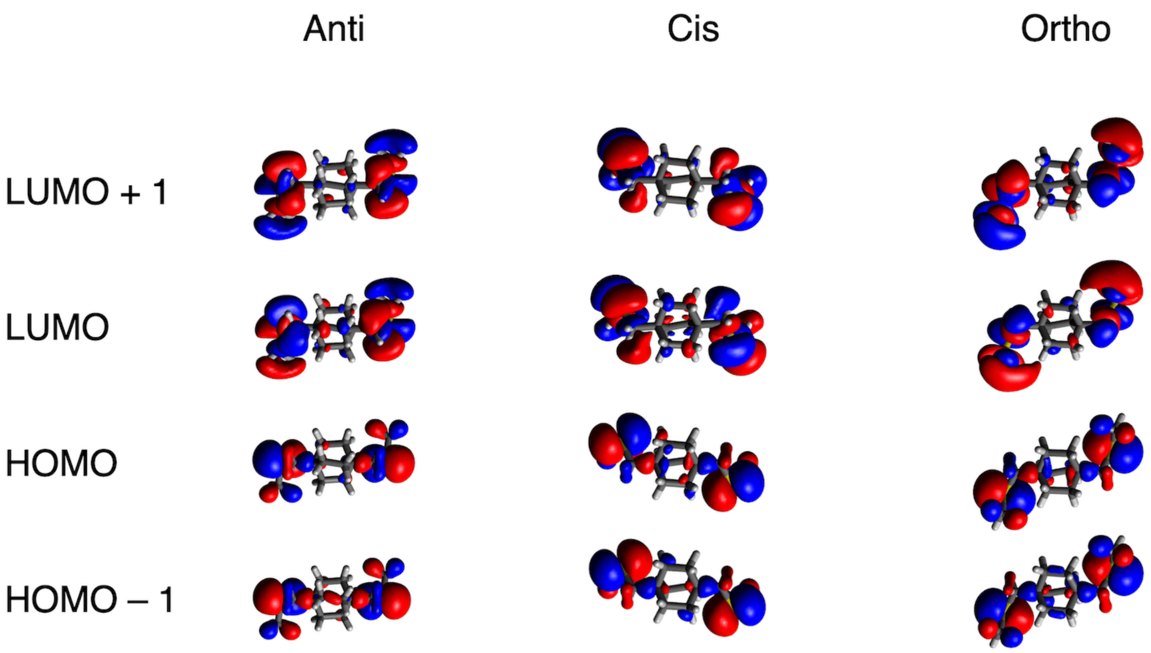

Figure S7. Frontier MOs of C222-H.

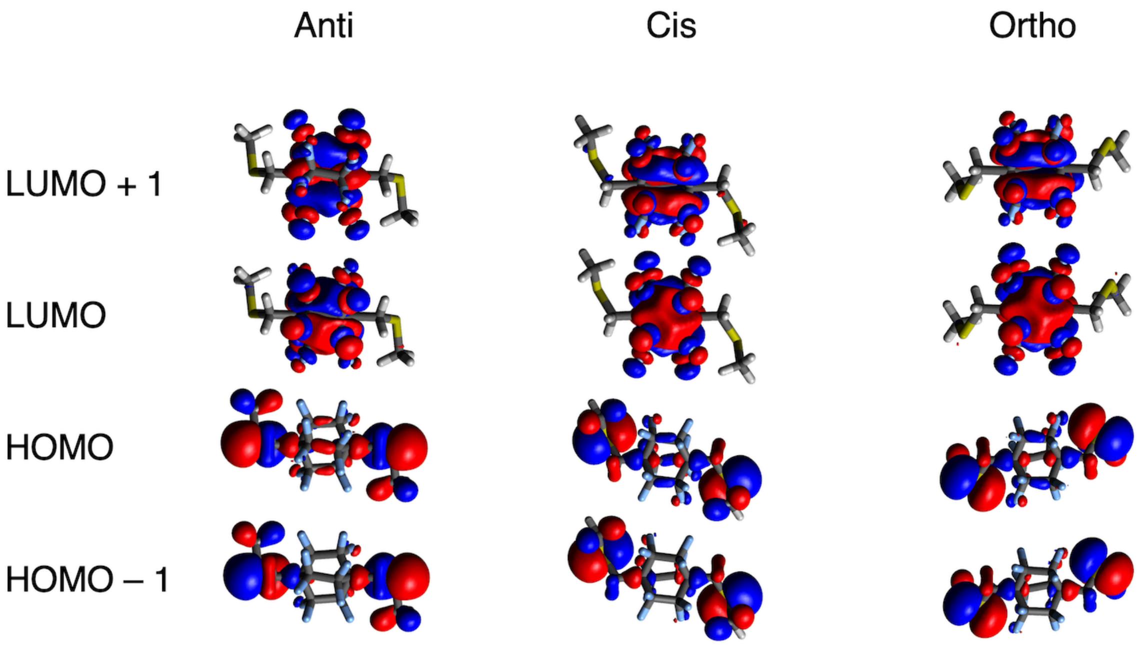

Figure S8. Frontier MOs of C222-F.

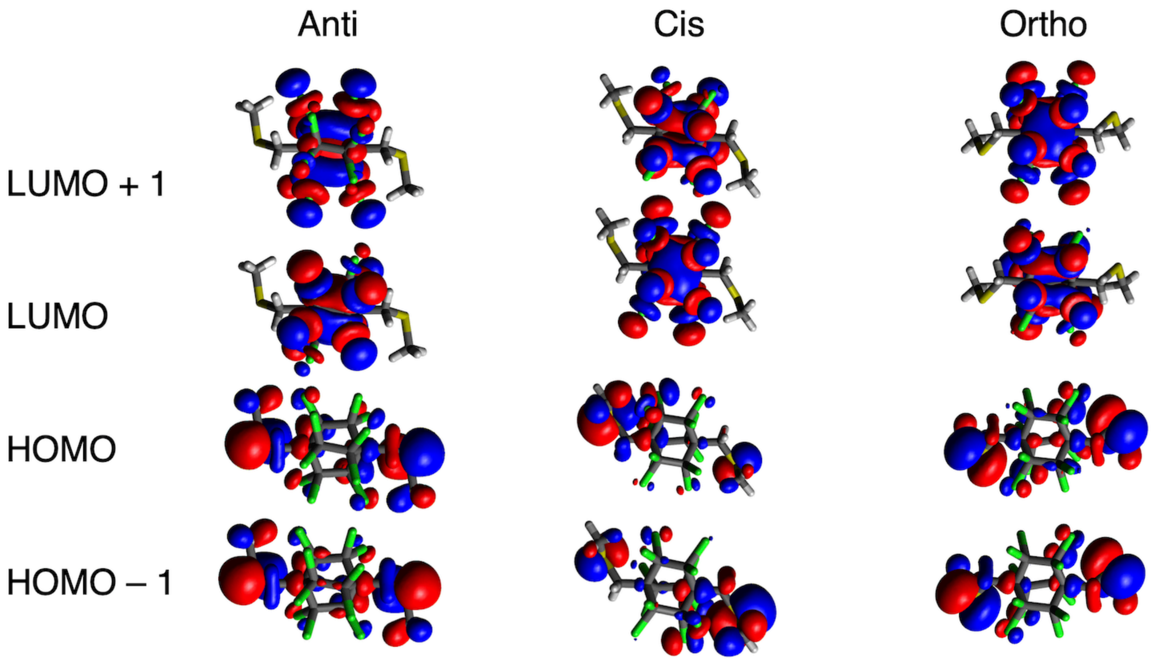

Figure S9. Frontier MOs of C222-Cl.

REFERENCES

1. Landrum, G. *Rdkit: Open-source cheminformatics*, http://www.rdkit.org.
2. Garner, M. H.; Koerstz, M.; Jensen, J. H.; Solomon, G. C., The Bicyclo[2.2.2]octane Motif: A Class of Saturated Group 14 Quantum Interference Based Single-Molecule Insulators. *J. Phys. Chem. Lett.* **2018,** *9* (24), 6941-6947.
3. Rappe, A. K.; Casewit, C. J.; Colwell, K. S.; Goddard, W. A.; Skiff, W. M., UFF, a full periodic table force field for molecular mechanics and molecular dynamics simulations. *J. Am. Chem. Soc.* **1992,** *114* (25), 10024-10035.
4. Casewit, C. J.; Colwell, K. S.; Rappe, A. K., Application of a universal force field to main group compounds. *J. Am. Chem. Soc.* **1992,** *114* (25), 10046-10053.
5. George, C. B.; Ratner, M. A.; Lambert, J. B., Strong Conductance Variation in Conformationally Constrained Oligosilane Tunnel Junctions. *J. Phys. Chem. A* **2009,** *113* (16), 3876-3880.
6. Li, H.; Garner, M. H.; Shangguan, Z.; Zheng, Q.; Su, T. A.; Neupane, M.; Li, P.; Velian, A.; Steigerwald, M. L.; Xiao, S.; Nuckolls, C.; Solomon, G. C.; Venkataraman, L., Conformations of cyclopentasilane stereoisomers control molecular junction conductance. *Chem. Sci.* **2016,** *7* (9), 5657-5662.
7. Garner, M. H.; Li, H.; Chen, Y.; Su, T. A.; Shangguan, Z.; Paley, D. W.; Liu, T.; Ng, F.; Li, H.; Xiao, S.; Nuckolls, C.; Venkataraman, L.; Solomon, G. C., Comprehensive suppression of single-molecule conductance using destructive σ-interference. *Nature* **2018,** *558* (7710), 415-419.
8. Li, H.; Garner, M. H.; Shangguan, Z.; Chen, Y.; Zheng, Q.; Su, T. A.; Neupane, M.; Liu, T.; Steigerwald, M. L.; Ng, F.; Nuckolls, C.; Xiao, S.; Solomon, G. C.; Venkataraman, L., Large Variations in the Single-Molecule Conductance of Cyclic and Bicyclic Silanes. *J. Am. Chem. Soc.* **2018,** *140* (44), 15080-15088.
9. Hanwell, M. D.; Curtis, D. E.; Lonie, D. C.; Vandermeersch, T.; Zurek, E.; Hutchison, G. R., Avogadro: an advanced semantic chemical editor, visualization, and analysis platform. *Journal of Cheminformatics* **2012,** *4* (1), 17.
10. http://avogadro.cc/ *Avogadro: an open-source molecular builder and visualizationtool. Version 1.2.0*, 2020.
