# Supplementary material for: Substituent Control of σ-Interference Effects in the Transmission of Saturated Molecules
Source: ACS Phys Chem Au. 2022 Apr 14;2(4):282–8. doi: 10.1021/acsphyschemau.2c00016 (PMC9955259; doi:10.1021/acsphyschemau.2c00016)

Substituent numbers

		C222	Si222	Ge222
	H	0	0	0
Me	CH_3	1	1	1
Et	CH_2CH_3		2	2
Vinyl	$CH=CH_2$	3	8	8
Ethynyl	C≡CH	4	9	9
	SiH_3		10	10
	OH	6	13	13
	OMe	7	14	14
	F	9	11	11
	Cl	10	12	12
	CF_3		6	6
	C≡N	8	15	
Ph	Phenyl		16	16

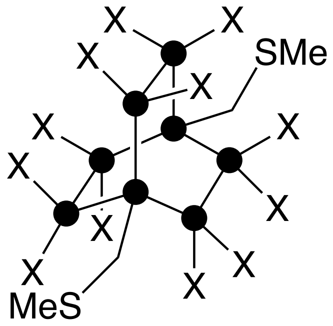

Supplement: Supplementary file 2 — pg2c00016_si_002.zip [file pg2c00016_si_002.zip › structures/substituent_numbers.pdf]
